# Impact of disease volume on survival efficacy of triplet therapy for metastatic hormone-sensitive prostate cancer: a systematic review, meta-analysis, and network meta-analysis

**DOI:** 10.1007/s10147-024-02485-4

**Published:** 2024-04-06

**Authors:** Akihiro Matsukawa, Pawel Rajwa, Tatsushi Kawada, Kensuke Bekku, Ekaterina Laukhtina, Jakob Klemm, Benjamin Pradere, Keiichiro Mori, Pierre I. Karakiewicz, Takahiro Kimura, Piotr Chlosta, Shahrokh F. Shariat, Takafumi Yanagisawa

**Affiliations:** 1 Department of Urology, Comprehensive Cancer Center, Medical University of Vienna, Wahringer Gurtel 18-20, 1090 Vienna, Austria; 2 Department of Urology, The Jikei University School of Medicine, Tokyo, Japan; 3 Department of Urology, Medical University of Silesia, Zabrze, Poland; 4 Department of Urology, Dentistry and Pharmaceutical Sciences, Okayama University Graduate School of Medicine, Okayama, Japan; 5 Institute for Urology and Reproductive Health, Sechenov University, Moscow, Russia; 6 Department of Urology, University Medical Center Hamburg-Eppendorf, Hamburg, Germany; 7 Department of Urology, La Croix Du Sud Hospital, Quint Fonsegrives, France; 8 Cancer Prognostics and Health Outcomes Unit, Division of Urology, University of Montreal Health Center, Montreal, Canada; 9 Department of Urology, Jagiellonian University, Medical College, Krakow, Poland; 10 Hourani Center for Applied Scientific Research, Al-Ahliyya Amman University, Amman, Jordan; 11 Department of Urology, University of Texas Southwestern Medical Center, Dallas, TX USA; 12 Department of Urology, Second Faculty of Medicine, Charles University, Prague, Czech Republic; 13 Department of Urology, Weill Cornell Medical College, New York, NY USA; 14 Karl Landsteiner Institute of Urology and Andrology, Vienna, Austria

**Keywords:** Androgen receptor signaling inhibitor, Docetaxel, Metastatic hormone-sensitive prostate cancer, High-volume, Low-volume

## Abstract

**Background:**

Triplet therapy, androgen receptor signaling inhibitors (ARSIs) plus docetaxel plus androgen-deprivation therapy (ADT), is a novel guideline-recommended treatment for metastatic hormone-sensitive prostate cancer (mHSPC). However, the optimal selection of the patient most likely to benefit from triplet therapy remains unclear.

**Methods:**

We performed a systematic review, meta-analysis, and network meta-analysis to assess the oncologic benefit of triplet therapy in mHSPC patients stratified by disease volume and compare them with doublet treatment regimens. Three databases and meeting abstracts were queried in March 2023 for randomized controlled trials (RCTs) evaluating patients treated with systemic therapy for mHSPC stratified by disease volume. Primary interests of measure were overall survival (OS). We followed the PRISMA guideline and AMSTAR2 checklist.

**Results:**

Overall, eight RCTs were included for meta-analyses and network meta-analyses (NMAs). Triplet therapy outperformed docetaxel plus ADT in terms of OS in both patients with high-(pooled HR: 0.73, 95%CI 0.64–0.84) and low-volume mHSPC (pooled HR: 0.71, 95%CI 0.52–0.97). There was no statistically significant difference between patients with low- vs. high-volume in terms of OS benefit from adding ARSI to docetaxel plus ADT (*p* = 0.9). Analysis of treatment rankings showed that darolutamide plus docetaxel plus ADT (90%) had the highest likelihood of improved OS in patients with high-volume disease, while enzalutamide plus ADT (84%) had the highest in with low-volume disease.

**Conclusions:**

Triplet therapy improves OS in mHSPC patients compared to docetaxel-based doublet therapy, irrespective of disease volume. However, based on treatment ranking, triplet therapy should preferably be considered for patients with high-volume mHSPC while those with low-volume are likely to be adequately treated with ARSI + ADT.

**Supplementary Information:**

The online version contains supplementary material available at 10.1007/s10147-024-02485-4.

## Introduction

The treatment landscape of metastatic hormone-sensitive prostate cancer (mHSPC) is rapidly changing [1–4]. Adding an androgen receptor signaling inhibitor (ARSI) with or without docetaxel to androgen deprivation therapy (ADT) is the current guideline-recommended therapy for mHSPC [5]. A recent meta-analysis showed a survival benefit of triplet therapy, consisting of ARSI plus docetaxel plus ADT, compared to ARSI or docetaxel-based doublet regimens; this benefit seemed more prominent in patients with high-volume disease [2]. Metastatic disease burdens, such as high- and low-volume disease, as proposed in the CHAARTED trial, have been widely adopted in clinical trial design, guidelines, and daily practice as it affects the disease state as well as biological and clinical behavior of the heterogeneity in mHSPC [1, 5–7]. Recently, results from subgroup analysis stratified by disease volume have been reported with comparative hazard ratios (HRs) for overall survival (OS) between patients with high- and low-volume mHSPC [8]. However, due to the small number of patients resulting in low statistical power due to the small number of patients, the efficacy of triplet therapy in patients with low-volume disease remains inconclusive [2, 8]. Therefore, we conducted this updated systematic review, meta-analysis, and network meta-analysis (NMA) to analyze OS effect of triplet therapy and compare its efficacy to doublet regimens in mHSPC patients according to disease volume.

## Methods

The protocol has been registered in the International Prospective Register of Systematic Reviews database (PROSPERO: CRD 404191). This meta-analysis and NMA were conducted based on the guidelines of the Preferred Reporting Items for Systematic reviews and Meta-Analyses (PRISMA) statement and AMSTAR2 checklist (Supplementary Table 1 and Supplementary Appendix 1) [9, 10].

### Search strategy

A literature search on PubMed^®^, Web of Science^™^, and Scopus^®^ databases was carried out to identify studies investigating the oncologic outcomes of systemic therapy for mHSPC in March 2023. The detailed search strategy is shown in Supplementary Appendix 2. We also looked for updates on ongoing trials and unpublished randomized controlled trials (RCTs) in abstracts presented at recent major conferences including the American Society of Clinical Oncology (ASCO) and the European Society for Medical Oncology (ESMO). The primary outcome of interest was OS. Initial screening based on the titles and abstracts was done by two investigators to identify eligible studies. Potentially relevant studies were subjected to a full-text review. To find additional studies of interest, manual searches of reference lists of relevant articles were also carried out. Disagreements were settled by consensus with co-authors.

### Inclusion and exclusion criteria

Studies were considered eligible if they examined patients with mHSPC (Patients), who were treated with triplet therapy (Interventions) and compared to those treated with other currently available treatment regimens (Comparisons), to assess the differential effects of treatment on OS stratified by disease volume (Outcome) only in RCTs (Study design). Studies lacking original patient data, reviews, letters, editorial comments, replies from authors, case reports, and articles not written in English were excluded. In cases of duplicate cohorts, the higher quality or the most recent publication was selected. References of all papers included were scanned for additional studies of interest.

### Data extraction

Data were extracted independently by two authors. The first author’s name, publication year, inclusion criteria, agents, agent dosage, number of patients, patient age, the number of patients with de novo disease, the number of patients stratified by disease volume, number of patients treated with docetaxel, and follow-up periods were extracted. Subsequently, the HRs and 95% confidence intervals (CIs) from Cox regression models for OS were retrieved. All discrepancies were resolved by consensus with co-authors.

### Risk of bias assessment

Assessment of study quality and risk of bias was carried out using the Cochrane Handbook for Systematic Reviews of Interventions risk-of-bias tool (RoB version 2) (Supplementary Fig. 1) [11]. The risk-of-bias assessment of each study was performed independently by two authors.

### Statistical analyses

#### Meta-analysis

Forest plots with HRs were utilized to analyze the association between systemic therapy and OS. Subgroup analyses were performed in the patients with high- vs. low-volume disease. An additional analysis was conducted in patients with de novo mHSPC. High-volume disease was defined in the CHAARTED trial as the presence of visceral metastases, or four or more bone metastases, of which at least one must be located outside the vertebral column or pelvic bone [7, 12]. Because of homogeneity across international phase 3 RCTs, fixed-effect model was used for calculations of HRs[13]. Heterogeneity among the outcomes of included studies in this meta-analysis was assessed using Cochrane’s Q test. All analyses were conducted using R version 4.2.2 (R Foundation for Statistical Computing, Vienna, Austria), and the statistical significance level was set at *P* < 0.05.

#### Network meta-analysis

A network meta-analysis using random-effect models with a frequentist approach was carried out for direct and indirect treatment comparisons [14, 15]. In the assessment of OS, contrast-based analyses were applied with estimated differences in the log HR and the standard error calculated from the published HR and 95%CIs [16]. The relative effects were presented as HRs and 95% CIs [14]. In addition, we estimated the relative ranking of the different treatments for each outcome using the surface under the cumulative ranking (SUCRA) [14]. Network plots were utilized to illustrate the connectivity of the treatment networks in terms of OS. All statistical analyses were performed using R version 4.2.2 (R Foundation for Statistical Computing, Vienna, Austria).

## Results

### Study selection and characteristics

**Fig. 1:**
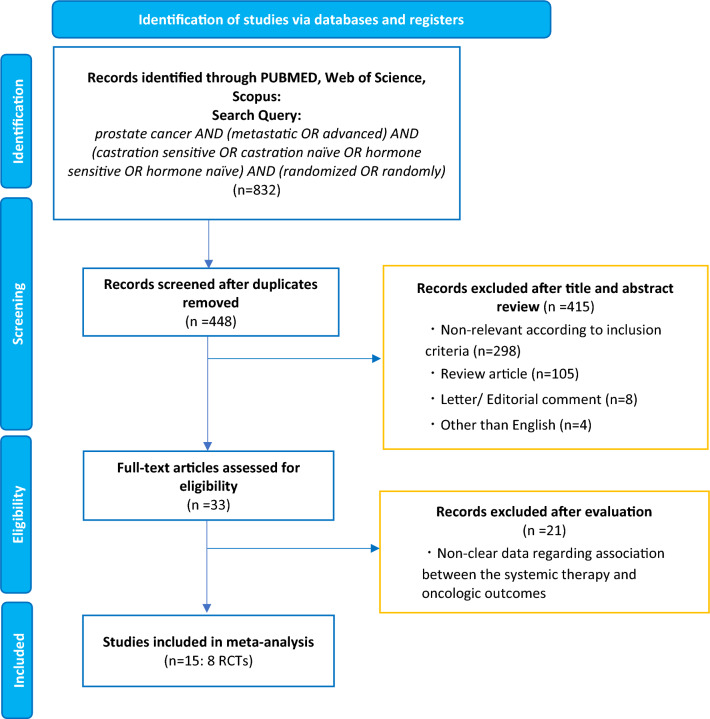
The Preferred Reporting Items for Systematic Reviews and Meta-analyses (PRISMA) flow chart, detailing the article selection process

Our initial search identified 832 records. After removing duplicates, 448 records remained for screening of titles and abstracts (Fig. 1). After screening, 415 articles were excluded, and a full-text review of 33 articles/abstracts was performed. According to our inclusion criteria, we finally identified eight RCTs (15 publications and abstracts) comprising 6969 patients eligible for meta-analyses and NMAs [3, 4, 6–8, 12, 17–25]. In the ARCHES, ENZAMET, and TITAN trials assessing the efficacy of ARSIs plus ADT combinations compared to ADT alone, patients were allowed to use docetaxel at the time of or after randomization[18, 26, 27]. However, only updated results from the ENZAMET trial provided the OS data on patients treated with or without docetaxel, stratified by disease volume[19]. Therefore, we excluded the ARCHES and TITAN trials. The demographics of each included study are shown in Table 1.

**Table 1:** Study demographics and oncologic outcomes of 8 RCTs included in the analyses

Study name/first author	Year	Treatment arm	Control arm	No. of pts			De novo mets., %		High-volume, %		HR for OS (95%CI)		Median F/U mo
				Total	T	C	T	C	T	C	High-volume	Low-volume	
ARASENS Smith et al Hussain et al	2022	DAR + DOC + ADT	DOC + ADT	1306	c	655	86	87	76	78	0.69 (0.57–0.82)	0.68 (0.41–1.13)	43
PEACE-1 Fizazi et al	2022	ABI + DOC + ADT	DOC + ADT	710	355	355	100	100	63	65	0.72 (0.55–0.95)	0.83 (0.50–1.38)	45.7
ENZAMET Davis et al	2019/2022	*ENZ + DOC + ADT	*DOC + ADT	503	254	249	71	73	71	72	0.87 (0.65–1.17)	0.61 (0.33–1.10)	68
		*ENZ + ADT	*ADT	622	310	312	ND	ND	39	39	0.69 (0.49–0.97)	0.51 (0.35–0.75)	
LATITUDE Fizazi et al	2017/2019	ABI + ADT	ADT	1199	597	602	100	100	82	78	0.62 (0.52–0.74)	0.72 (0.47–1.1)	51.8
STAMPEDE (Arm G) James et al Hoyle et al	2017/2019	ABI + ADT	ADT	990	493	497	94	96	54	51	0.6 (0.46–0.78)	0.64 (0.42–0.97)	40
STAMPEDE (Arm B,C,E) James et al Clarke et al	2016/2019	DOC + ADT	ADT	1086	362	724	96	95	54	57	0.81 (0.64–1.02)	0.76 (0.54–1.07)	78.2
CHAARTED Sweeny et al Kyriakopoulos et al	2015/2018	DOC + ADT	ADT	790	397	393	73	73	66	64	0.63 (0.5–0.79)	1.04 (0.7–1.55)	53.7
GETUG-AFU15 Gravis et al	2013/2016	DOC + ADT	ADT	385	192	193	68	66	48	47	0.78 (0.56–1.09)	0.72 (0.47–1.1)	83.9

*RCT* Randomized controlled trial, *T* Treatment arm, *C* Control arm, *No* Number, *Pts* Patients, *HR* Hazard ratio, *OS* Overall survival, *CI* Confidence interval, *F/U* Follow-up, *DOC* Docetaxel, *ADT* Androgen deprivation therapy, *ABI* Abiraterone acetate, *APA* Apalutamide, *DAR* Darolutamide, *ENZ* Enzalutamide, *mHSPC* metastatic hormone-sensitive prostate cancer, *ND* No data

^*^Data on patients treated with docetaxel was extracted. Control arm contained ADT + nonsteroidal antiandrogen

^**^Described as rates in overall cohort

### Assessment of Risk of bias and quality of study

The risk of bias judgments of each domain for each included study is summarized in Supplementary Fig. 1. All included studies had a low risk of bias owing to the nature of prospective phase 3 RCTs. The quality assessment of this meta-analysis was performed according to the AMSTAR2 checklist; overall confidence in the results of this review was “High” (Supplementary Appendix 1) [10].

### Meta‑analysis of triplet therapy vs. docetaxel plus ADT

**Fig. 2:**
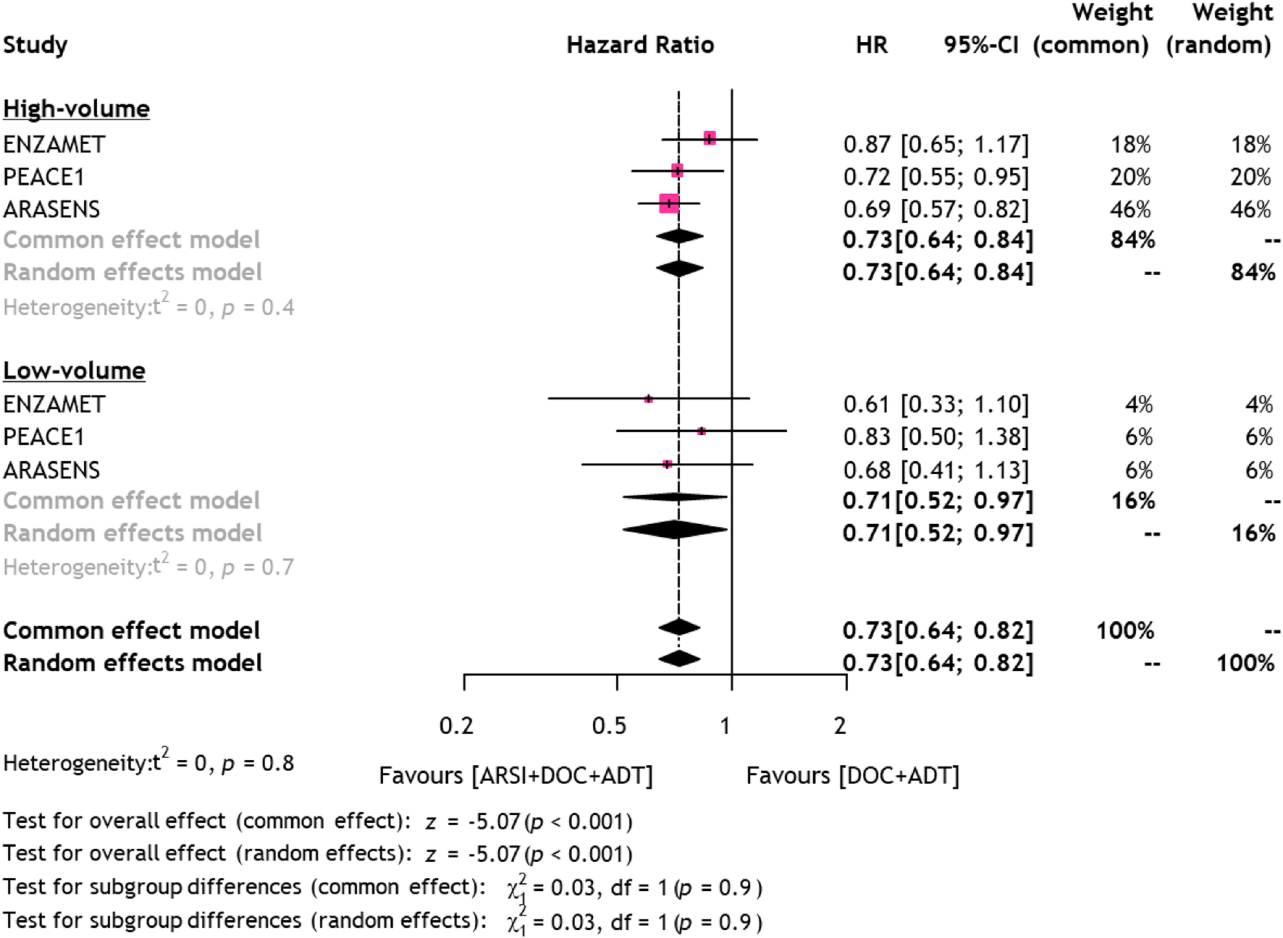
Forest plots showing the association between OS and triplet or docetaxel-based doublet therapy for mHSPC stratified by disease volume *OS* Overall survival, *mHSPC* metastatic hormone-sensitive prostate cancer, *ARSI* Androgen receptor signaling inhibitors, *DOC* Docetaxel, *ADT* Androgen deprivation therapy

Three studies comprising 2519 patients provided data on OS in mHSPC patients treated with triplet therapy or docetaxel plus ADT separately for high- and low-volume disease. As shown in Fig. 2, adding ARSI to docetaxel plus ADT improved OS in both patients with high- (pooled HR: 0.73, 95%CI 0.64–0.84) and low- (pooled HR: 0.71, 95%CI 0.52–0.97) volume disease. There was no statistically significant difference between patients with low- vs. high-volume in terms of OS benefit from adding ARSI to docetaxel plus ADT (*p* = 0.9).

In an analysis focusing on patients with de novo mHSPC, adding ARSI to docetaxel plus ADT improved OS in patients with high-volume disease (pooled HR: 0.72, 95%CI 0.62–0.83), but not in those with low-volume disease (pooled HR: 0.74, 95%CI 0.53–1.01). There was no significant difference in OS between patients with low- vs. high-volume in terms of OS benefit from adding ARSI to docetaxel plus ADT (*p* = 0.9; Supplementary Fig. 3). The Cochrane’s Q tests revealed no significant heterogeneity in the analysis.

### Network meta‑analysis of overall survival among currently available systemic therapies

All eight RCTs including seven different regimens were eligible for this NMA to compare the OS of currently available systemic treatment regimens. The networks of eligible comparisons are graphically described as network plots addressing OS (Supplementary Fig. 2).

#### Patients with high-volume disease

**Fig. 3:**
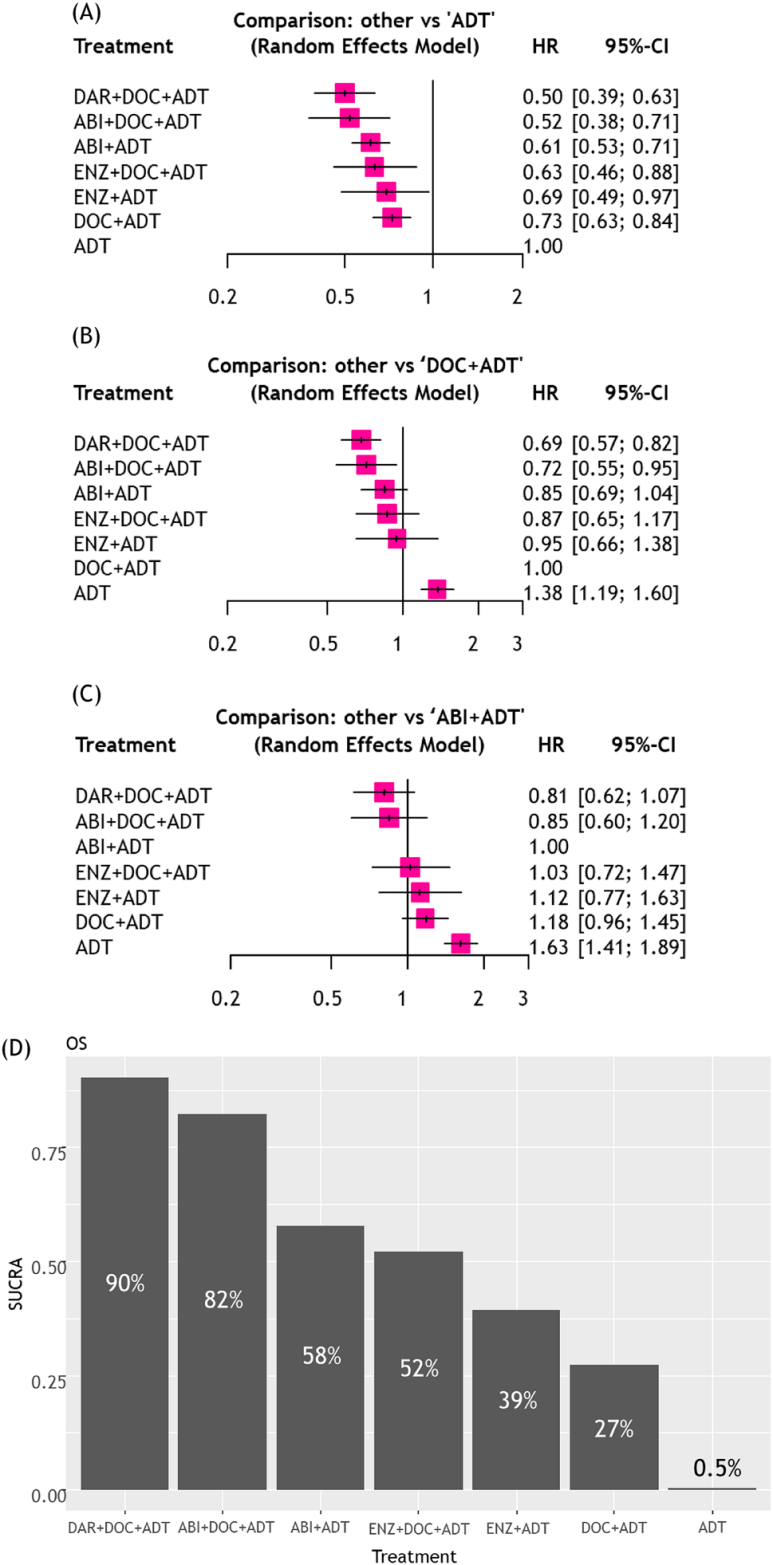
Results of NMAs for OS in mHSPC patients with “high-volume” disease treated with systemic therapies; **A** Forest plots (ADT alone as a comparator), **B** Forest plots (ABI + ADT as a comparator), **C** Treatment ranking based on SUCRA graph *NMA* Network meta-analysis, *OS* Overall survival, *mHSPC* metastatic hormone-sensitive prostate cancer, *ADT* Androgen deprivation therapy, *ABI* Abiraterone, *APA* Apalutamide, *ARSI* Androgen receptor signaling inhibitors, *ENZ* Enzalutamide, *DAR* Darolutamide, *DOC* Docetaxel

As shown in Fig. 3, all combinations significantly improved OS compared to ADT alone. Compared to docetaxel plus ADT, darolutamide plus docetaxel plus ADT (HR: 0.69, 95%CI 0.57–0.82) and abiraterone plus docetaxel plus ADT (HR: 0.72, 95%CI 0.55–0.95) improved OS. Compared to abiraterone plus ADT, triplet therapy using darolutamide plus docetaxel plus ADT (HR: 0.81, 95%CI 0.62–1.07) and abiraterone plus docetaxel plus ADT (HR: 0.85, 95%CI 0.60–1.20) did not reach statistical significance in terms of improved OS. Based on the SUCRA analysis of treatment rankings for OS, darolutamide plus docetaxel plus ADT (90%) had the highest likelihood of providing the maximal OS benefit, followed by abiraterone plus docetaxel plus ADT (82%).

#### Patients with low-volume disease

**Fig. 4:**
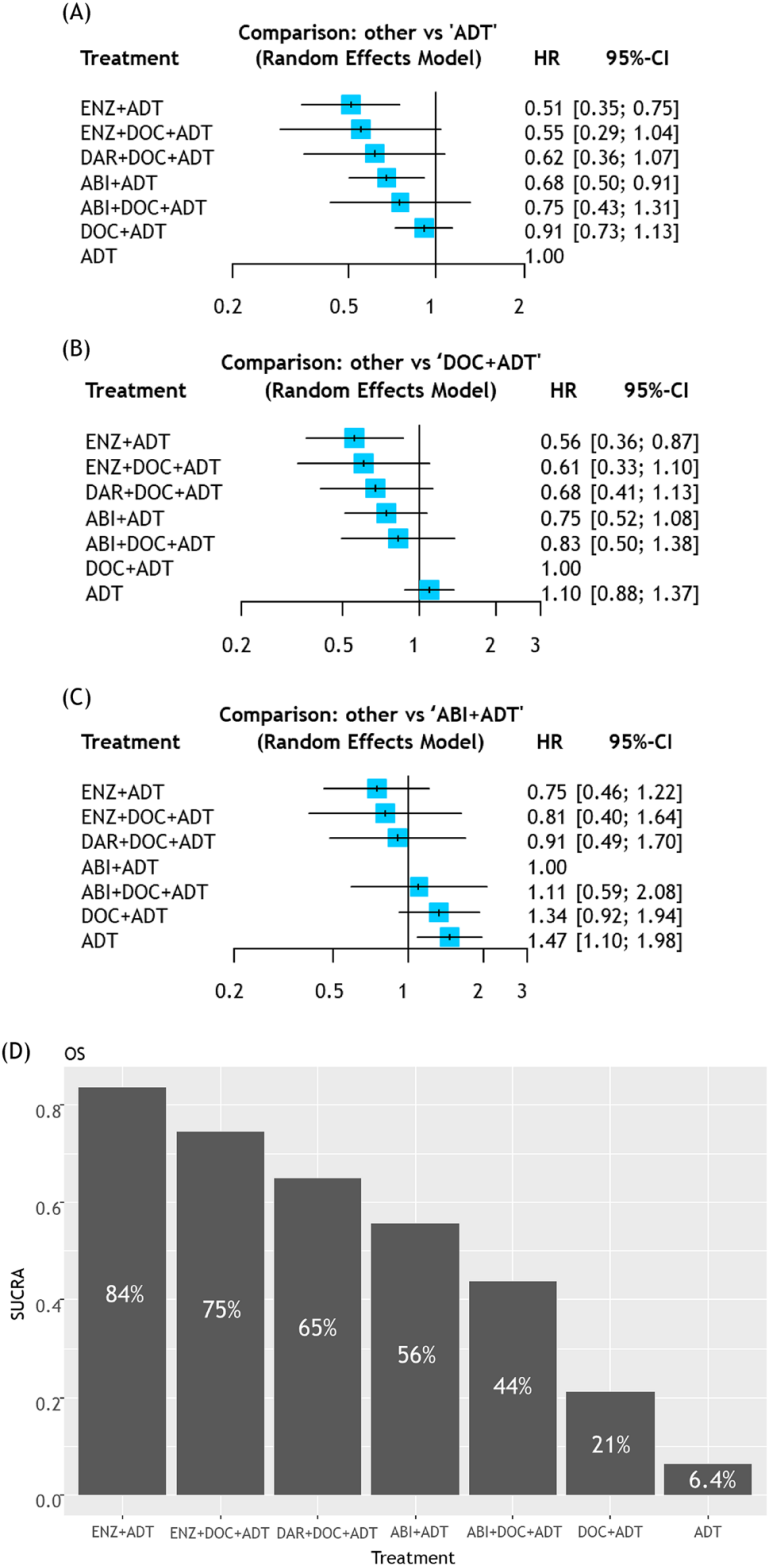
Results of NMAs for OS in mHSPC patients with “low-volume” disease treated with systemic therapies; **A** Forest plots (ADT alone as a comparator), **B** Forest plots (ABI + ADT as a comparator), **C** Treatment ranking based on SUCRA graph *NMA* Network meta-analysis, *OS* Overall survival, *mHSPC* metastatic hormone-sensitive prostate cancer, *ADT* Androgen deprivation therapy, *ABI* Abiraterone, *APA* Apalutamide, *ARSI* Androgen receptor signaling inhibitors, *ENZ* Enzalutamide, *DAR* Darolutamide, *DOC* Docetaxel

As shown in Fig. 4, compared to ADT alone, enzalutamide plus ADT (HR: 0.51, 95%CI 0.35–0.75) and abiraterone plus ADT (HR: 0.68, 95%CI 0.50–0.91) improved OS. Despite some evidence to the contrary, all triplet combinations did not reach the conventional level of statistical significance. In addition, compared to docetaxel plus ADT, only enzalutamide plus ADT significantly improved OS (HR: 0.56, 95%CI 0.36–0.87). Considering comparison to abiraterone plus ADT doublet, none of the other combination therapies showed significant improvements. Based on the SUCRA analysis of treatment rankings for OS, enzalutamide plus ADT had the highest likelihood of providing the maximal OS benefit (84%), followed by enzalutamide plus docetaxel plus ADT (75%) and darolutamide plus docetaxel plus ADT (65%).

## Discussion

We report an updated meta-analysis and NMA analyzing and comparing the effect of triplet therapy on OS in patients with mHSPC stratified by disease volume. There are several key findings to our study. First, triplet therapy outperformed docetaxel plus ADT in terms of OS in patients with both high- and low-volume disease. Second, treatment ranking analysis confirmed that triplet therapy had the highest likelihood of improved OS among currently available treatment regimens in patients with high-volume disease. Conversely, in low-volume mHSPC, doublet therapy using ARSI (enzalutamide) plus ADT had the highest likelihood of improved OS.

Our meta-analysis demonstrated the OS benefit from triplet therapy even in mHSPC patients with low-volume disease compared to docetaxel plus ADT. Indeed, HRs for OS from each RCT in patients with low-volume disease were all comparable with those with high-volume, while no study showed statistical significant difference potentially due to the low number of patients and events for low-volume disease resulting in a low statistical power (Fig. 4) [3, 8, 19]. Based on this and a previous meta-analysis without the results from a subgroup analysis of the ARASENS trial, ARSI-based doublet therapy is currently recommended for low-volume disease [2, 28]. However, when synthesizing the results from three RCTs, we demonstrated significant OS benefit from triplet therapy compared to docetaxel plus ADT, even in patients with low-volume disease. On the contrary, when focusing on patients with de novo low-volume disease, we found no significant OS benefit from triplet therapy compared to docetaxel plus ADT. A possible explanation is low statistical power based on the low population of this group, highlighting the need for further studies with larger sample sizes to confirm these findings.

Low-volume mHSPC generally has a more favorable disease trajectory and relatively good prognosis when treated with combination therapies; therefore, long-term follow-up with larger sample size is needed to elucidate the survival outcomes. A recent meta-analysis with long-term follow-up (median 6 years) using individual participant data from the GETUG-15, CHAARTED, and STAMPEDE trials showed OS benefit from adding docetaxel to ADT in mHSPC patients, irrespective of disease volume, while the absolute value was more prominent in patients with high- (HR 0.60, 95%CI 0.52–0.68) compared to those with low- (HR 0.78, 95%CI 0.64–0.94) volume disease [29]. Notably, despite including updated results from the ENZAMET trial, which has a median follow-up of 68 months, the follow-up periods in the ARASENS and PEACE-1 trials at 45.7 and 43 months respectively, were not long enough to fully evaluate the OS outcomes in patients with low-volume disease [3, 4, 19]. Extended follow-up periods are needed to confirm a robust OS benefit from triplet therapy in patients with low-volume disease and to identify the best combination for each individual patient.

Our treatment rankings revealed that triplet therapy, darolutamide plus docetaxel plus ADT, had the highest likelihood of improved OS in patients with high-volume disease. This updated result, including subgroup analyses from the ENZAMET and ARASENS trials, was in line with the results from a previous NMA [2]. High-volume disease is generally associated with biologically and clinically aggressive disease bearing a high probability of harboring androgen receptor-independent cells; suggesting a possible rationale for the efficacy of adding cytotoxic chemotherapy to ADT + ARSI for high-volume disease [30]. In agreement with this concept, the OS benefit from adding docetaxel to ADT was most prominent in patients with high-volume disease in the CHAARTED trial [21]. Taken together, the state of current evidence suggests that triplet therapy with docetaxel plus ASRI plus ADT should be considered in patients with high-volume disease who can tolerate it.

In contrast, enzalutamide plus ADT not only had the highest likelihood of improved OS in patients with low-volume mHSPC but also significantly improved OS when compared to docetaxel plus ADT alone. This suggests a limited role for triplet therapy in patients with low-volume disease and it supports individual shared decision-making. However, our analysis should be interpreted with caution due to possible selection bias. In this NMA, we extracted the data on subgroups treated with or without docetaxel from the PEACE-1 and ENZAMET trials. In these trials, the decision of docetaxel application was based on the physician’s discretion. This might have led to selection bias; younger, healthier patients with more aggressive diseases are more likely to receive docetaxel. For example, in the ENZAMET trial, patients who did not receive docetaxel were more likely to harbor low-volume disease; 71% of patients treated with docetaxel harbored high-volume disease, while this rate was only 37% in patients who did not receive docetaxel [19].

Since the publication of the CHAARTED trial results in 2015, docetaxel plus ADT has gained acceptance as a standard of care (SOC) in patients with mHSPC. As mentioned before, this greatly affected the study design/eligibility of RCTs [18, 26, 31]. Unlike the ENZAMET trial, the ARCHES and TITAN trials also allowed the use of docetaxel as SOC; patients treated with docetaxel were included in both treatment arms. This made reliable NMAs including “true” ARSI-based doublet regimens difficult. Therefore, our treatment rankings lack the apalutamide plus ADT with or without docetaxel, not reflecting all the currently available regimens.

In the argument of whether triplet therapy outperforms ARSI-based doublet therapy in terms of survival outcomes, our previous NMA showed that darolutamide plus docetaxel plus ADT outperformed abiraterone plus ADT in terms of OS in the overall cohort[2]. However, present NMAs stratified by disease volume failed to show the statistical OS superiority of triplet therapy over ARSI-based doublet therapy in both patients with high- and low volume disease. A possible explanation is that these results were based on subgroup analyses from each RCT, limiting the statistical power. Only a head-to-head RCT comparing triplet therapy versus ARSI-based doublet therapy will clarify the potential impact on survival outcomes.

Last but not least, the benefit-harm balance is, indeed, an important factor for clinical decision-making. A recent meta-analysis showed that ARSI-based doublet therapy had high probabilities for a net clinical benefit; however, docetaxel-based doublet as well as triplet therapy appeared unlikely to be beneficial [32]. Docetaxel is known to increase the risk of severe adverse events and can reduce the patient quality of life [2, 33]. Our previous meta-analysis confirmed that docetaxel-based combination had a higher likelihood of severe adverse events, with triplet therapies being the highest adverse event rates [2, 33]. In the subgroup analysis of the ARASENS trial, 74% of patients with low-volume disease who received triplet therapy experienced severe adverse events [8]. Although our study showed the oncologic utility of triplet therapy, shared decision-making considering the benefit-harm balance is essential, specifically in such patients with a long life expectancy [8]. Further investigation is needed to select the optimal candidates who are most likely to benefit from triplet therapy.

Besides the abovementioned issues, the current study has several limitations that need to be considered. First, despite careful data extraction from RCTs, each study differed in patient proportion, such as the rates of de novo*/*metachronous and high/low-volume disease. Our analyses based on subgroup analyses of each RCT might reduce the patient heterogeneity; however, this differential proportion could potentially affect the outcomes. Second, in the ARCHES, ENZAMET, and TITAN trials assessing the efficacy of ARSIs plus ADT combinations compared to ADT alone, patients were allowed to use docetaxel at the time of or after randomization [18, 26, 27]. However, only updated results from the ENZAMET trial provided the OS data on patients treated with or without docetaxel, stratified by disease volume [19]. Therefore, we excluded the ARCHES and TITAN trials. Third, NMAs have a limited role in facilitating patient selection; this approach cannot substitute for a direct comparison of each treatment and is mostly hypothesis-generating. Additionally, while patients with low-volume disease are generally less likely to experience mortality in the short-term follow-up, the effectiveness of triplet therapy may not be apparent in our analysis. Our findings need to be validated in head-to-head, well-designed RCTs. Fourth, our analyses are limited in assessing which regimens are the best combination for each clinical setting. Due to the nature of subgroup analyses, a limited number of studies assessing the outcomes stratified by disease volume. Furthermore, there was a paucity of studies reporting outcomes stratified by metachronous high- or low-volume separately. Fifth, although we investigated OS stratified by disease volume, other factors, such as Gleason pattern 5, TP53 mutations, also affect mortality even for patients with low-volume disease [34, 35]. Therefore, patients with these factors may benefit from triplet therapy even in the context of low-volume disease. Finally, the ENZAMET trial included the use of non-steroidal antiandrogen therapy with ADT in the control arm. This might provide a differential survival benefit in the control arm, therefore, weighing against the survival outcomes of enzalutamide. Again, we need further RCTs, especially for RCTs directly comparing triplet therapy versus ARSI-based doublet therapy. In addition, the comparative efficacy of darolutamide with other ARSIs as ARSI-based doublet combinations from the ongoing ARANOTE trial (NCT04736199) will enrich the treatment option for mHSPC. Based on this analysis and a previous meta-analysis, which did not include results from a subgroup analysis of the AR therapy is currently recommended for patients with low-volume disease [36].

## Conclusions

We found that compared to docetaxel plus ADT triplet therapy improves OS in patients with mHSPC, irrespective of disease volume. Treatment ranking revealed that triplet therapy should be a first-choice option in patients with high-volume disease who can tolerate it. However, in patients with low-volume disease, ARSI plus ADT doublet seemed to be the first choice with respect to OS. Further investigation with well-designed RCTs is awaited to clarify the comparative oncologic outcomes between triplet and ARSI-based doublet therapies and select the optimal candidates who are most likely to benefit from triplet therapy.

### Supplementary Information

Below is the link to the electronic supplementary material.

Supplementary file1 (PDF 1638 KB)

## Abbreviations

ADT: Androgen deprivation therapy
ASCO: American Society of Clinical Oncology
ARSI: Androgen receptor signaling inhibitor
CI: Confidence interval
ESMO: European Society for Medical Oncology
HR: Hazard ratio
mHSPC: Metastatic hormone-sensitive prostate cancer
NMA: Network meta-analysis
OS: Overall survival
PRISMA: Preferred Reporting Items for Systematic reviews and Meta-Analyses
RCTs: Randomized controlled trials
SOC: Standard of care
SUCRA: Surface under the cumulative ranking
